# Multistage reconstruction following extensive necrotizing fasciitis of the posterior trunk in a resource-limited setting: a case report

**DOI:** 10.1093/jscr/rjag759

**Published:** 2026-09-20

**Authors:** Emmanuel Girma, Einar Eriksen, Fikru Delesa, Filagot Bizuneh

**Affiliations:** Department of Surgery, Black Lion Comprehensive Specialized Hospital, MCM Comprehensive Specialized Hospital, Addis Ababa, Ethiopia; Children’s Burn and Wound Care Foundation, MCM Comprehensive Specialized Hospital, Bole Subcity, Addis Ababa, Ethiopia; Children’s Burn and Wound Care Foundation, MCM Comprehensive Specialized Hospital, Bole Subcity, Addis Ababa, Ethiopia; Department of Plastic Surgery, MCM Comprehensive Specialized Hospital, Bole Subcity, Addis Ababa, Ethiopia; Children’s Burn and Wound Care Foundation, MCM Comprehensive Specialized Hospital, Bole Subcity, Addis Ababa, Ethiopia; Department of Plastic Surgery, MCM Comprehensive Specialized Hospital, Bole Subcity, Addis Ababa, Ethiopia; Department of Plastic Surgery, MCM Comprehensive Specialized Hospital, Bole Subcity, Addis Ababa, Ethiopia

**Keywords:** necrotizing fasciitis, wound healing, skin grafting, plastic surgical reconstruction, extensive back wound, large wound defect

## Abstract

Necrotizing fasciitis (NF) is a life-threatening soft tissue infection requiring urgent debridement. Although infection control is paramount, timely reconstruction is equally critical to restore physiological stability and achieve durable closure. A 38-year-old woman with uncontrolled type 2 diabetes mellitus presented with extensive posterior trunk NF complicated by septic shock and diabetic ketoacidosis. Radical debridement created a 40 × 30 cm defect with exposed ribs, scapula, and thoracolumbar spinous processes. Reconstruction was staged. Rib corticotomy was performed to promote granulation, prominent spinous processes were reduced and covered with paraspinal muscle advancement, and definitive closure was achieved using two-stage 3:1 meshed split-thickness skin grafting. Postoperative management emphasized glycemic control, nutritional optimization, and pressure offloading. Complete graft take was achieved without donor-site complications. Staged reconstruction combined with structured postoperative care can achieve reliable closure in extensive posterior trunk NF, even in resource-limited settings.

## Introduction

Necrotizing fasciitis is a fulminant soft tissue infection characterized by rapid fascial necrosis, systemic toxicity, and high mortality, reported between 20% and 40% in contemporary series [1–4]. Mortality increases significantly in patients with delayed presentation, septic shock, and comorbidities such as diabetes mellitus [2, 5–7].

Urgent broad-spectrum antimicrobial therapy and radical surgical debridement are universally accepted as the cornerstones of treatment [1–3]. However, extensive excision frequently results in large soft tissue defects that cannot be closed primarily. Persistent open wounds contribute to sustained hypermetabolism, protein loss, fluid imbalance, and impaired immune function. Although the importance of early debridement is well established, the reconstructive phase of management, particularly in massive truncal disease—has received comparatively less emphasis.

We report a case of near-total posterior trunk necrotizing fasciitis managed with staged reconstruction and a structured perioperative protocol in a resource-limited setting, highlighting operative decision-making and the physiologic impact of timely wound coverage.

## Case presentation

A 38-year-old woman with a five-year history of untreated type 2 diabetes mellitus presented after two months of progressive back swelling, blistering, purulent discharge, and skin discoloration. She had declined pharmacologic therapy at diagnosis (HbA1c 10.9%). In the weeks prior to admission, she developed abdominal pain, tachypnea, fever, and altered mentation.

On presentation, she was acutely ill with blood pressure 105/65 mmHg, respiratory rate 34 breaths/min, temperature 38.5°C, oxygen saturation 92% on room air, and random blood glucose 448 mg/dL. Glasgow Coma Scale was 14/15 with confusion. Examination revealed extensive necrosis across the thoracic and lumbar back with bullae, purulence, and crepitus (Fig. 1).

**Figure 1:**
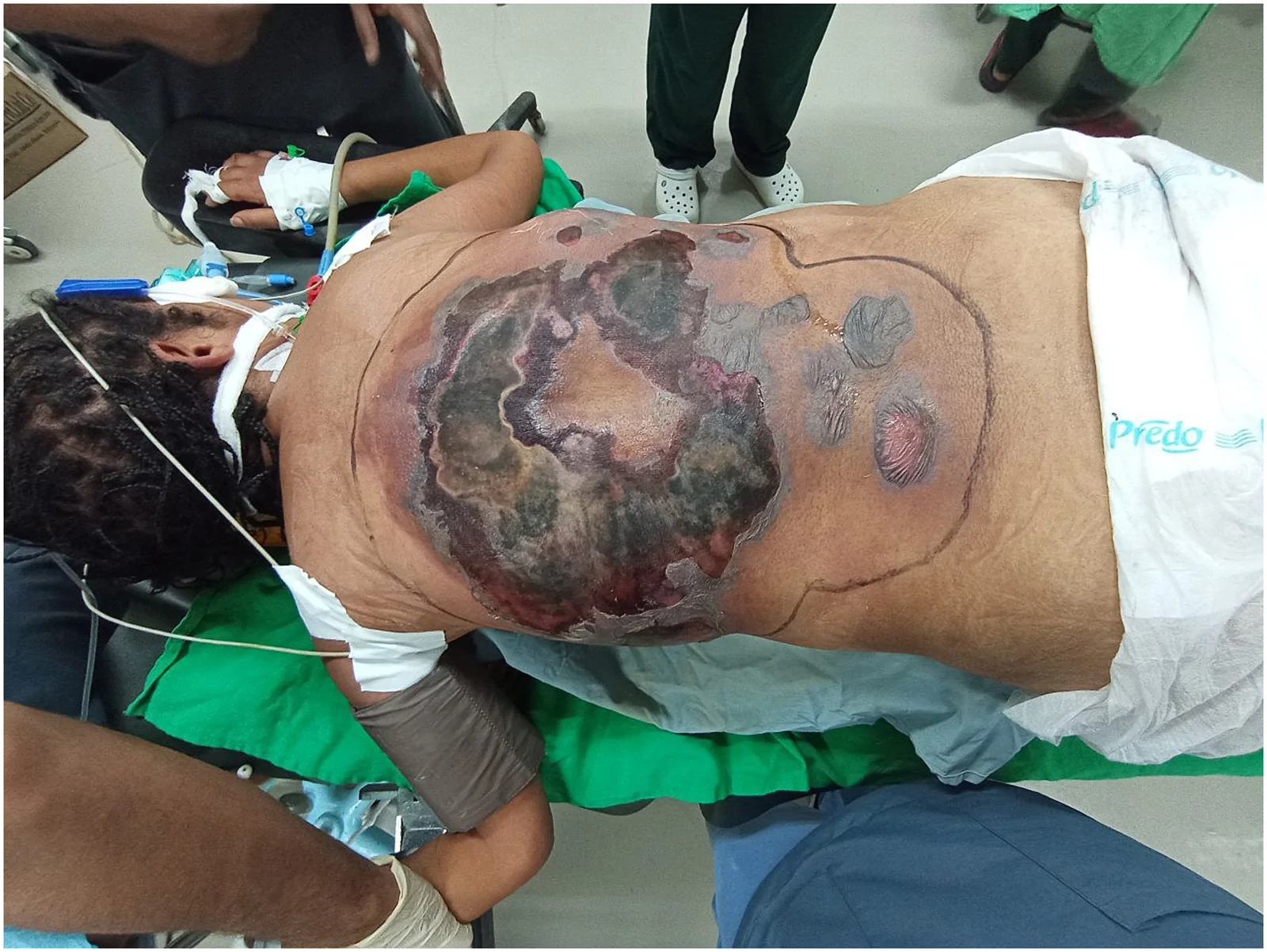
Pre-operative picture upon patient presentation.

Laboratory evaluation demonstrated leukocytosis, hemoglobin 7 g/dL, metabolic acidosis, and ketonemia consistent with diabetic ketoacidosis. Plain radiography showed subcutaneous gas. A diagnosis of necrotizing fasciitis complicated by sepsis and diabetic ketoacidosis was established.

After initiation of broad-spectrum intravenous antibiotics, insulin infusion, transfusion, and intensive care stabilization, emergency radical debridement was performed. Approximately 40 × 30 cm of necrotic skin, subcutaneous tissue, and muscle were excised to bleeding margins, leaving exposed posterior ribs, distal scapular tip, and thoracolumbar spinous processes (Fig. 2).

**Figure 2:**
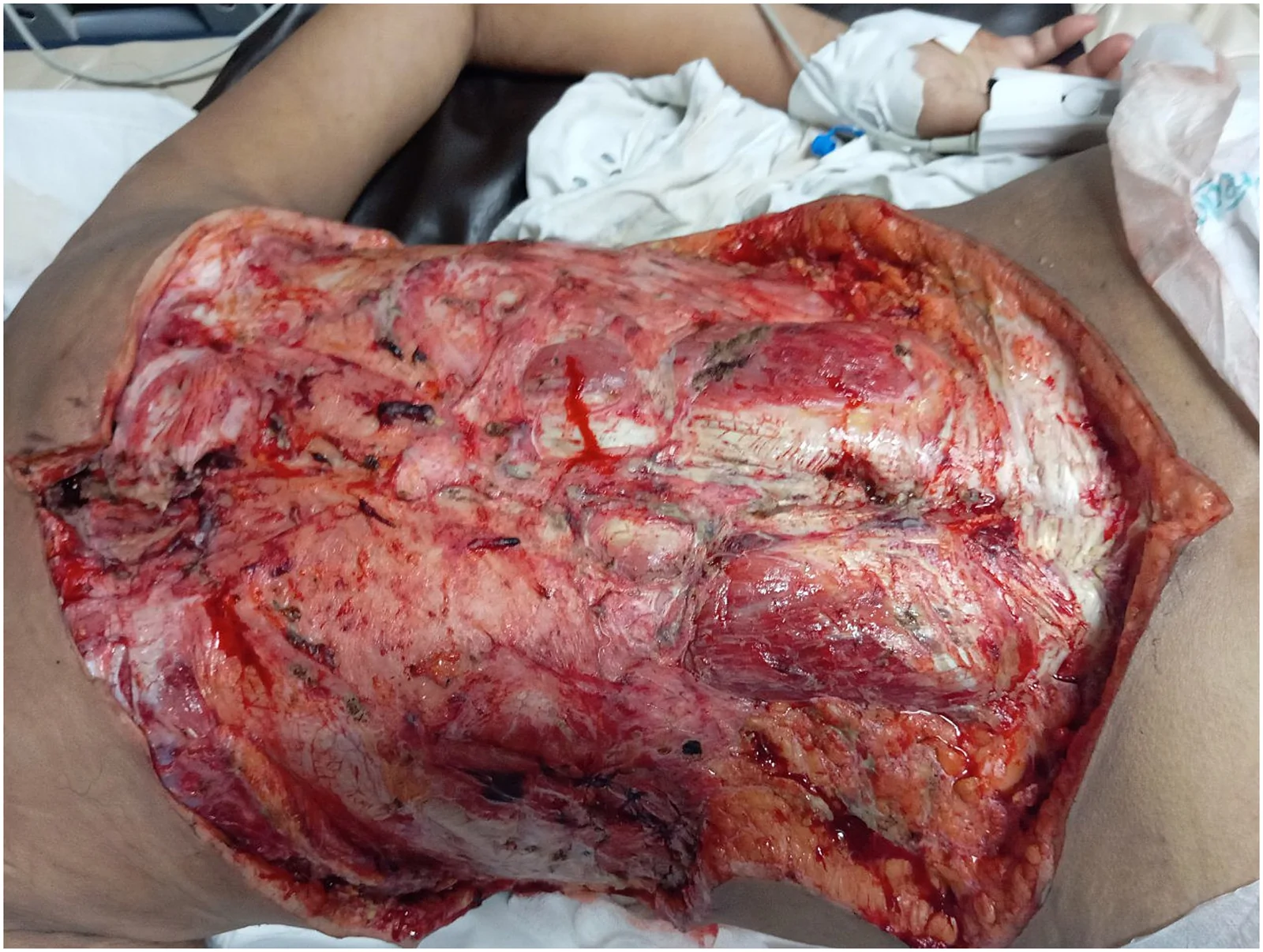
Post excision and debridement of all necrotic tissue.

A second-look debridement was undertaken following stabilization.

## Reconstructive strategy and operative technique

Given the patient’s physiologic instability and extensive exposed axial skeleton, immediate definitive coverage was deferred. A staged reconstructive approach was adopted to convert non-graftable surfaces into vascularized beds while minimizing operative stress.

Exposed posterior ribs were treated with multiple controlled cortical perforations to induce punctate bleeding and promote granulation. Prominent spinous process tips were selectively reduced using a bone chisel to eliminate desiccated cortex and decrease pressure risk. Adjacent paraspinal musculature was mobilized and advanced to provide vascularized coverage over viable bone where feasible. These maneuvers rendered previously non-graftable areas suitable for skin grafting.

Daily wound care under monitored procedural sedation facilitated thorough irrigation and mechanical debridement, allowing progressive granulation tissue formation across the defect (Fig. 3).

**Figure 3:**
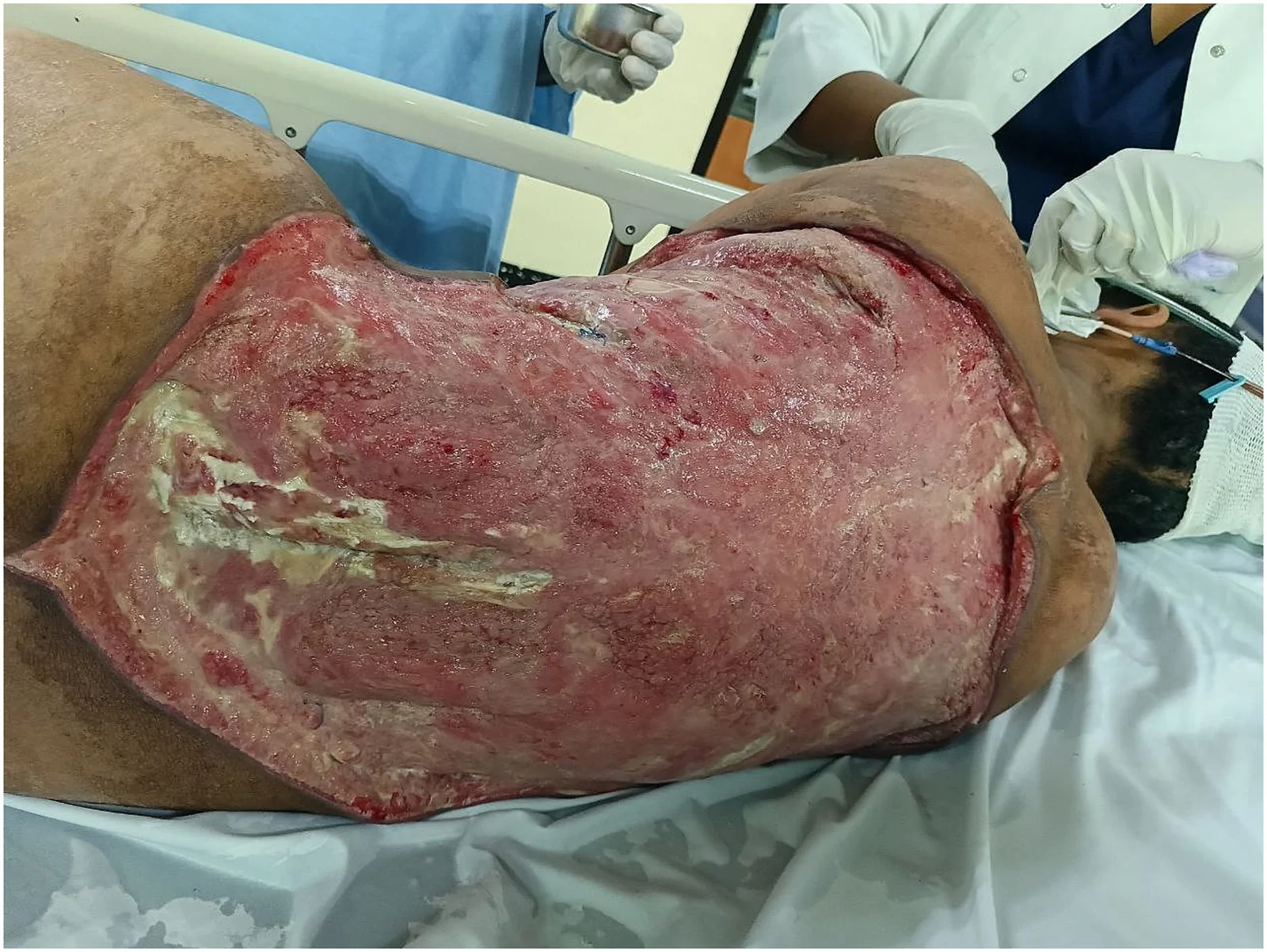
After wound has granulated well with dedicated wound care protocols in place.

One month after initial debridement, first-stage reconstruction was performed. Split-thickness skin grafts were harvested from the left thigh using a handheld Humby knife and meshed at 3:1. Limited advancement of adjacent soft tissue reduced defect dimensions prior to graft placement. Approximately 70% of the wound surface was grafted. The procedure was intentionally limited to reduce operative stress and preserve donor sites (Fig. 4).

**Figure 4:**
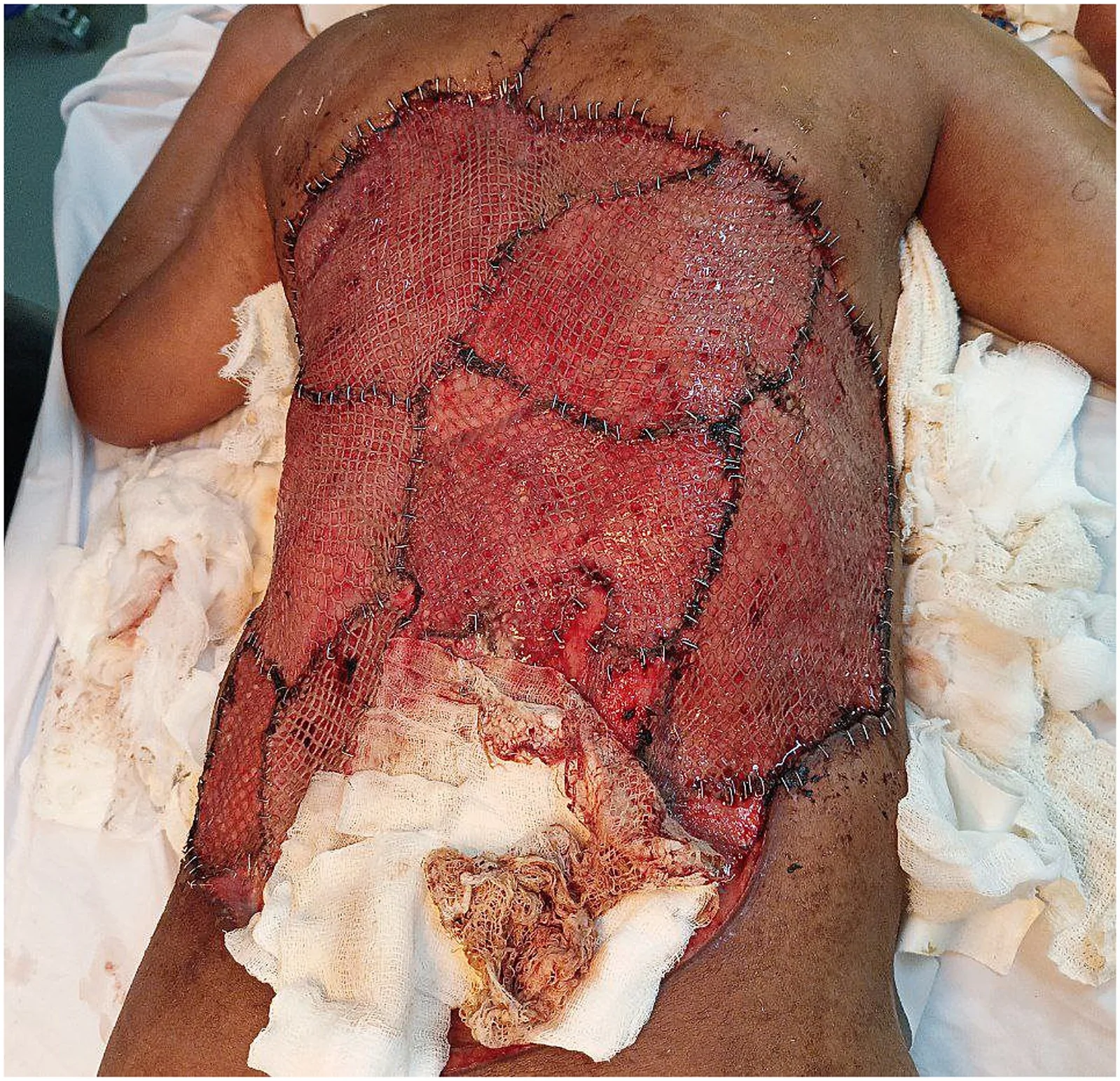
First post op day after skin graft surgery, undergoing wound dressing change.

Three weeks later, complete graft take and improved physiologic status were confirmed (Figs 5 and 6). Second-stage grafting of remaining small wound was performed using contralateral thigh donor sites (Fig. 7). Complete wound coverage was achieved (Figs 8 and 9).

**Figure 5:**
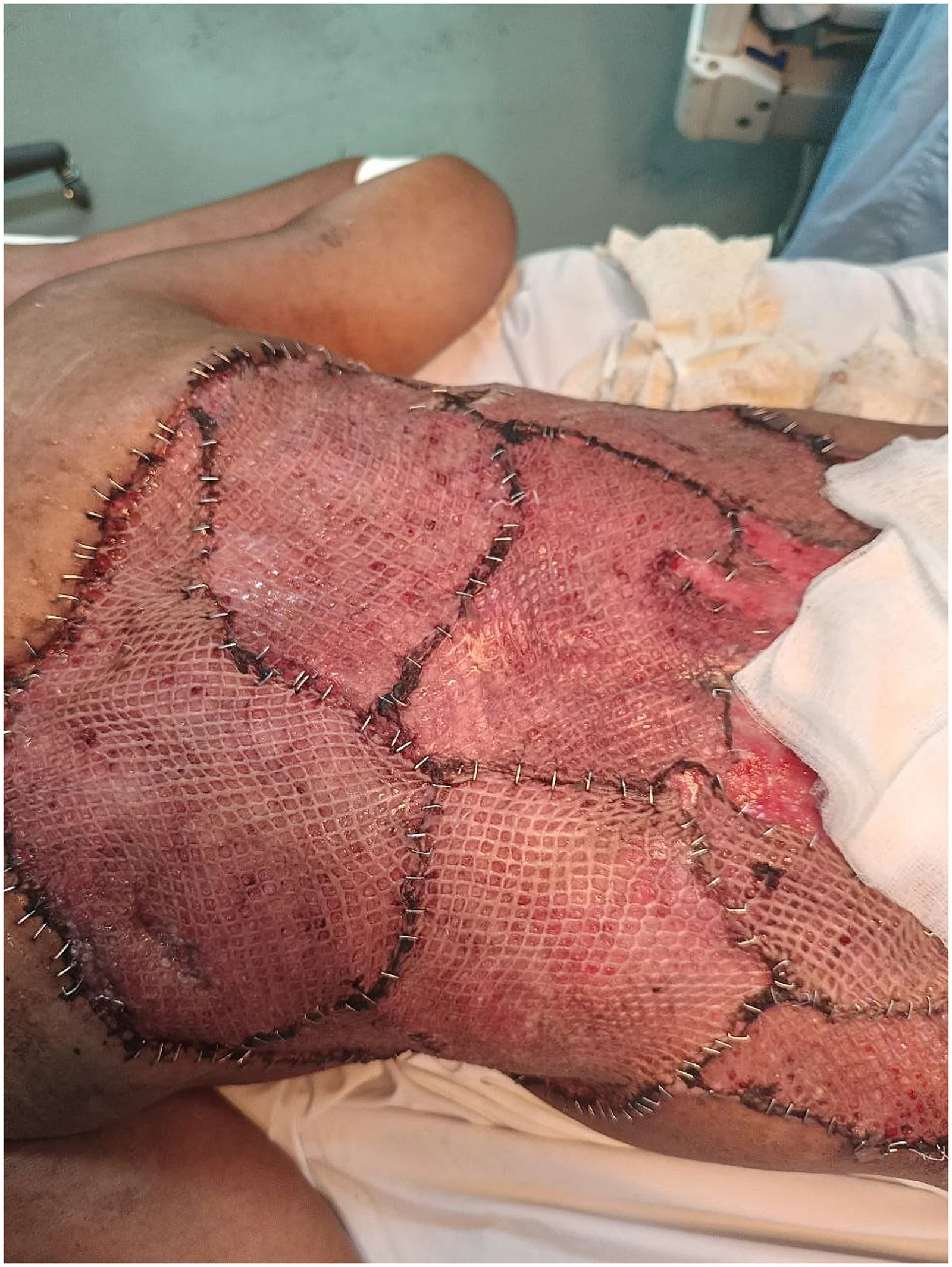
Fifth post op date after skin graft surgery, graft healed with complete take.

**Figure 6:**
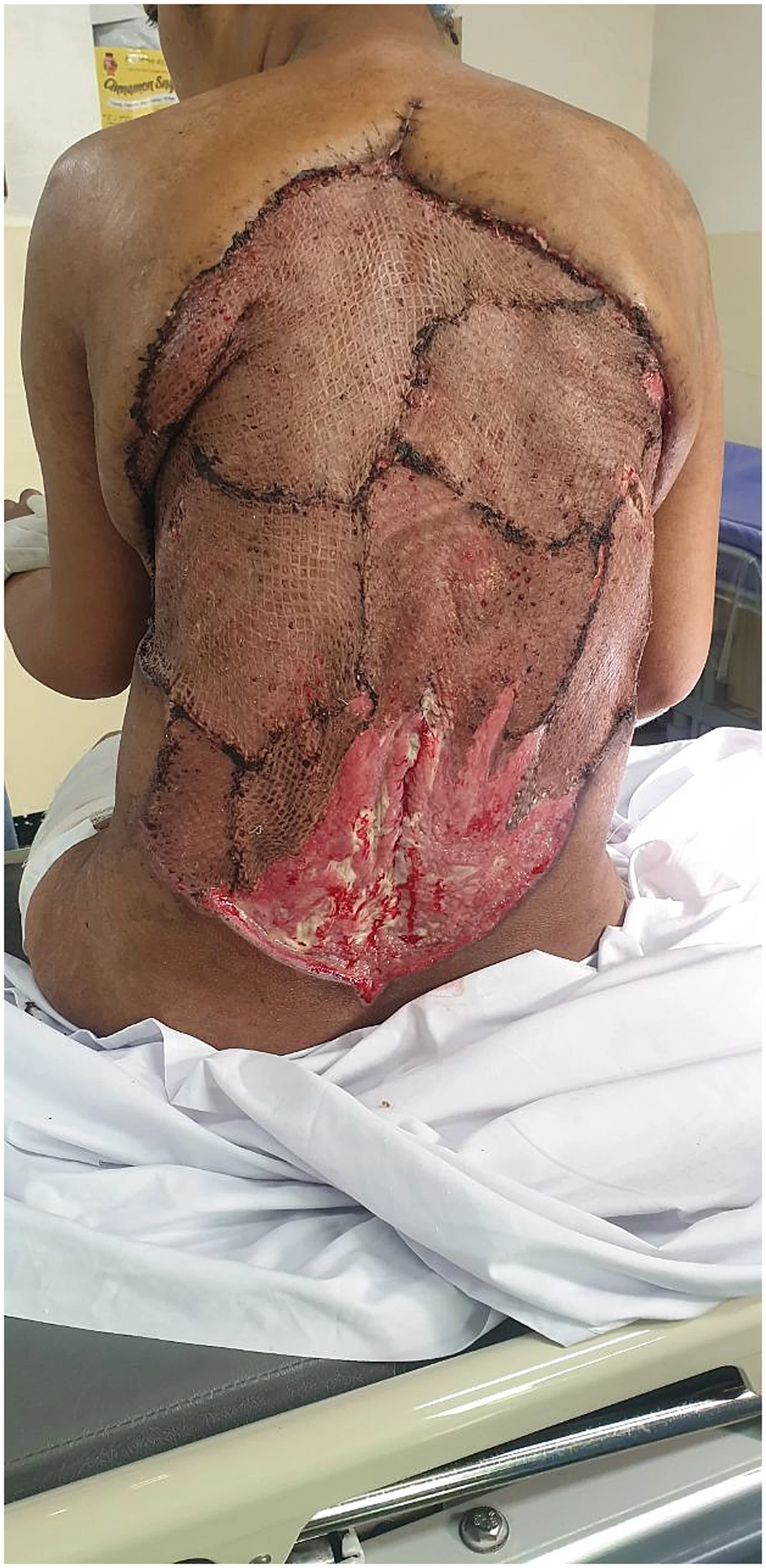
Eighth post op date after skin graft surgery, underwent staple removal, graft healed completely.

**Figure 7:**
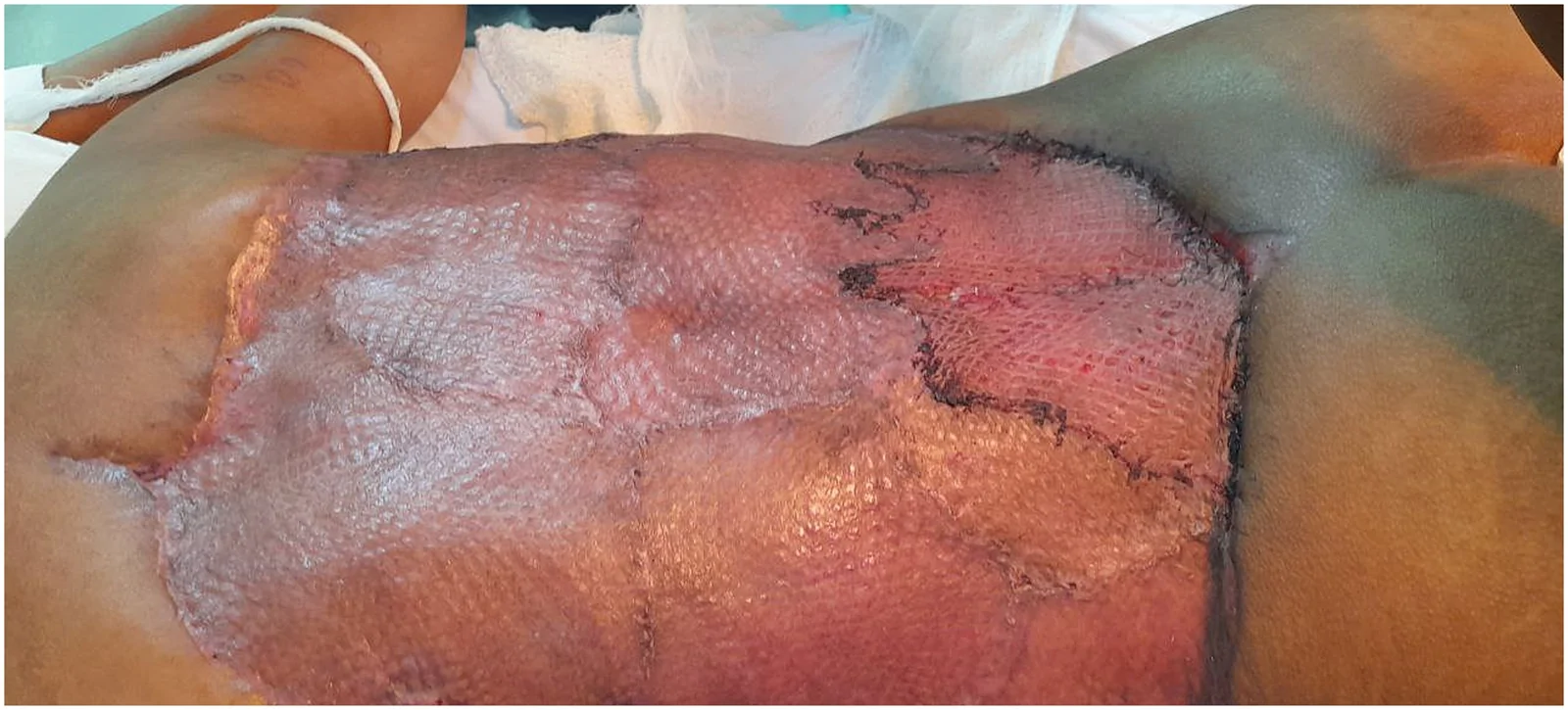
Fifth post op date after second round skin graft coverage surgery was done to cover remaining wounds. New graft take also confirmed.

**Figure 8:**
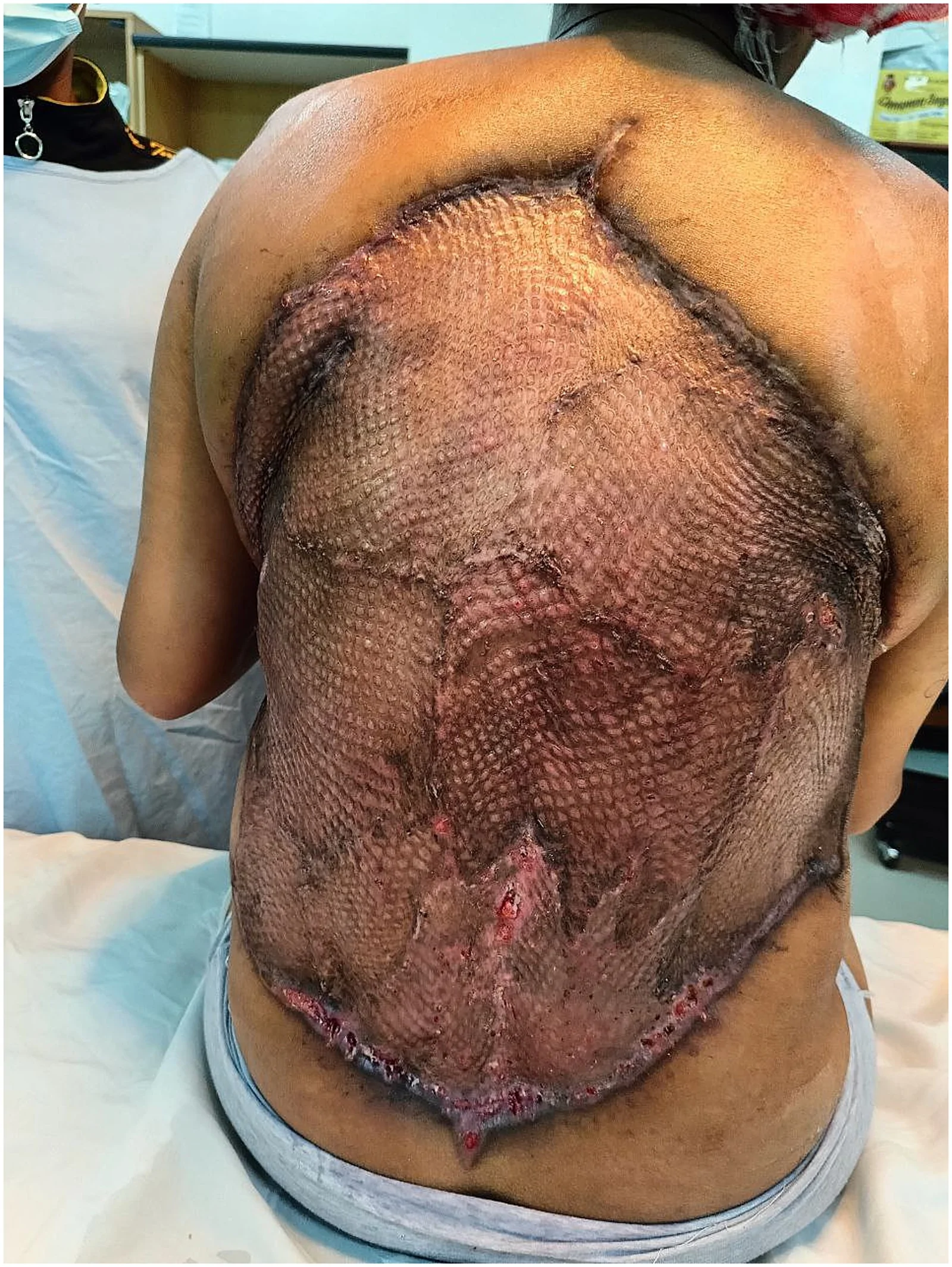
14th post op date after second skin graft surgery, all grafts have taken and continued healing taking place.

**Figure 9:**
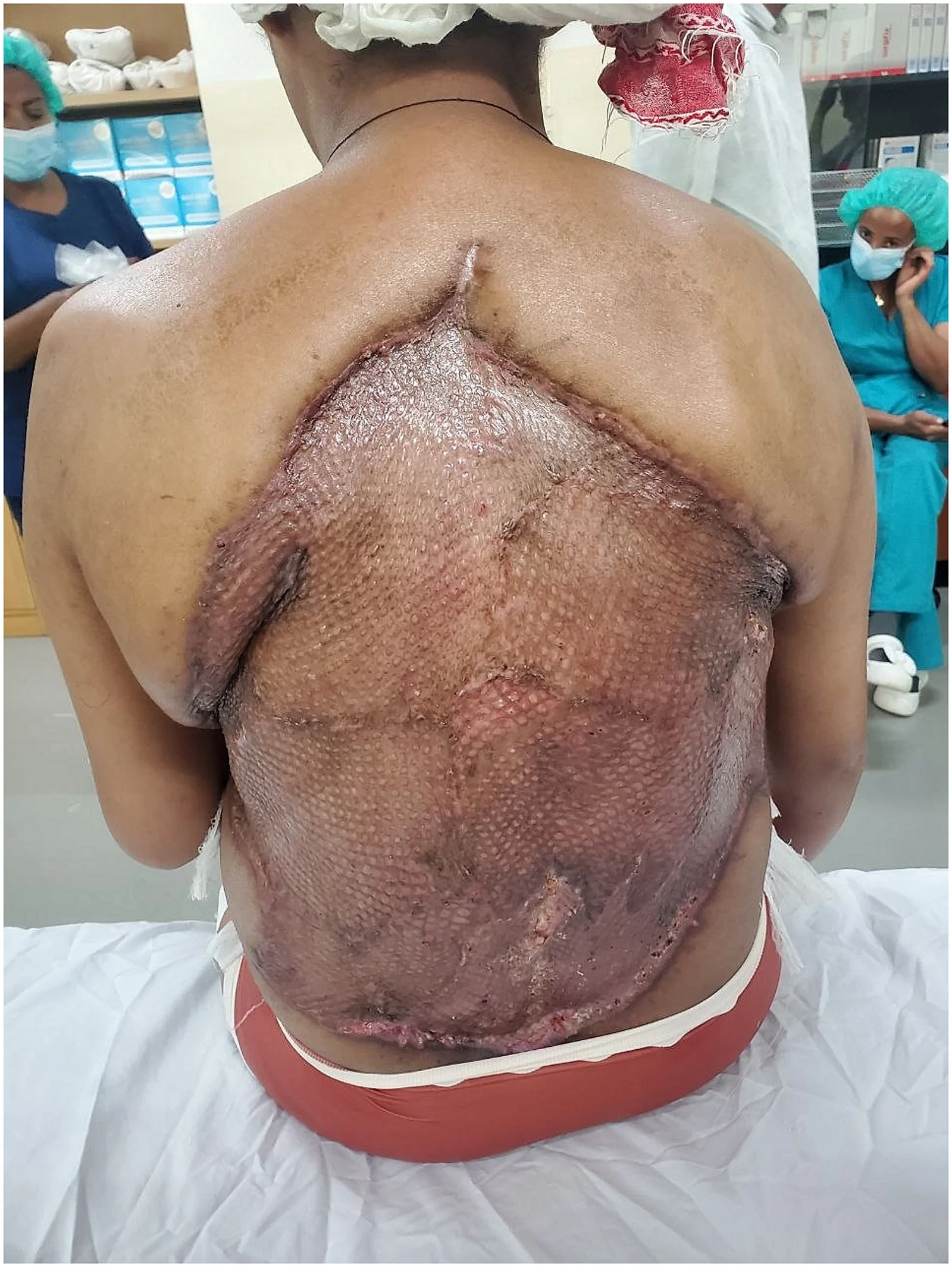
Skin grafts upon discharge of the patient, final cosmetic and functional outcome.

## Structured postoperative management

Postoperative success was closely linked to a structured unit protocol. Dressings were first opened on postoperative day one rather than day five, and thereafter changed daily under procedural sedation to permit meticulous cleansing, early hematoma detection, and precise graft assessment.

Strict pressure offloading was maintained using alternating lateral positioning with protective padding to prevent shear on posterior graft sites.

All patients in the unit receive mandatory nasogastric enteral feeding using a high-calorie blended formulation prepared by nursing staff (milk, bananas, boiled eggs, and sugar), providing ~1000 kcal per liter. Caloric requirements were calculated individually and administered in divided 3-h intervals. In this patient, uncontrolled diabetes necessitated modification of carbohydrate composition in collaboration with internal medicine and nutrition teams to achieve caloric goals without compromising glycemic control. Insulin therapy was titrated aggressively.

Following staged wound closure and metabolic optimization, the patient demonstrated progressive normalization of inflammatory markers, improved glycemic stability, and functional recovery. Complete graft take was achieved in both stages without donor-site morbidity.

## Discussion

Necrotizing fasciitis remains associated with substantial mortality despite advances in antimicrobial therapy and critical care [1–4]. Delayed presentation, diabetes mellitus, septic shock, and extensive truncal involvement are recognized adverse prognostic factors [2, 5–7].

While radical debridement is lifesaving, extensive open wounds perpetuate hypermetabolism and immune dysfunction. Timely reconstruction therefore plays a physiologically restorative role. Early involvement of plastic surgeons in necrotizing soft tissue infections has been associated with improved outcomes [8–11].

In this case, staged reconstruction balanced physiologic safety with definitive closure. Cortical perforation of exposed ribs and selective spinous process reduction with local muscle advancement were essential in converting skeletal exposure into graftable surfaces. Although flap reconstruction may be considered for extensive defects, operative duration, and physiologic burden may be prohibitive in critically ill patients. Staged meshed split-thickness grafting provided reliable coverage with reduced operative stress.

Structured postoperative management—including daily sedation-assisted wound care, strict glycemic control, nutritional optimization, and pressure offloading—was integral to achieving complete graft take and systemic recovery.

## Conclusion

In extensive posterior trunk necrotizing fasciitis, survival depends not only on early radical debridement but also on deliberate reconstructive planning and structured postoperative management. Staged split-thickness skin grafting following meticulous wound bed preparation, combined with coordinated multidisciplinary care, can achieve durable closure and physiologic recovery even in high-risk patients within resource-limited settings.

## Institutional approval

Permission to publish this case report was granted by MCM Comprehensive Specialized Hospital Institutional Review Board, the institution where the patient received treatment. The institution approved dissemination of the clinical data and images for scientific and educational purposes.

## References

[1] Wong CH, Chang HC, Pasupathy S et al. Necrotizing fasciitis: clinical presentation, microbiology, and determinants of mortality. J Bone Joint Surg Am 2003;85:1454–60. 10.2106/00004623-200308000-00005 12925624

[2] Anaya DA, Dellinger EP. Necrotizing soft-tissue infection: diagnosis and management. Clin Infect Dis 2007;44:705–10. 10.1086/511638 17278065

[3] Misiakos EP, Bagias G, Patapis P et al. Current concepts in the management of necrotizing fasciitis. Front Surg 2014;1:36. 10.3389/fsurg.2014.00036 PMC4286984 25593960

[4] Janis JE et al. Practical review of necrotizing fasciitis: principles and evidence-based management. Plast Reconstr Surg Glob Open 2024;12:e5533. 10.1097/GOX.0000000000005533 PMC10798703 38250213

[5] Mindaye ET, Terefe F. Management outcome and associated factors of necrotizing soft tissue infections in an Ethiopian tertiary care hospital. Ethiop J Health Sci 2024;34:365–72. 10.4314/ejhs.v34i5.4 PMC11811385 39944383

[6] Yilma Y, Fikadu Y, Belachew T. A five-year review of necrotizing fasciitis at Jimma University specialized hospital. Int J Med Med Sci 2016;8:145–9. 10.5897/IJMMS2016.1253

[7] Misiakos EP, Bagias G, Patapis P et al. Current concepts in the management of necrotizing fasciitis. *Front Surg* 2014;1:36. 10.3389/fsurg.2014.00036 PMC4286984 25593960

[8] Susini P, Marcaccini G, Efica J et al. Fournier’s gangrene surgical reconstruction: a systematic review. J Clin Med 2024;13:4085. 10.3390/jcm13144085 PMC11278345 39064124

[9] Torres García CD, Navarrete Espinosa DL, Figueroa Ramón ZS et al. Lower extremity necrotizing fasciitis managed with NPWT and split-thickness skin graft: a case report. Int J Med Sci Clin Res Stud 2022;02:724–30. 10.47191/ijmscrs/v2-i8-02

[10] Yokoyama A, Takase C. Impact of early surgical intervention of plastic surgeons on necrotizing soft tissue infection prognosis. Cureus 2021;13:e19382. 10.7759/cureus.19382 PMC8655320 34925985

[11] Flap coverage for necrotising soft tissue infections . A systematic review. Infect Dis (Auckl) 2021;53:128–40. 10.1016/j.burns.2021.01.005 34172327

